# Spatial transcriptomics: a bibliometric analysis with large language model on English literatures

**DOI:** 10.1093/bib/bbaf553

**Published:** 2025-10-26

**Authors:** Huiyang Li, Haixiao Wu, Wenjuan Ma, Shu Li, Jun Cai, Yile Lin, Jin Zhang, Yingmei Wang, Chao Zhang

**Affiliations:** Department of Gynecology and Obstetrics, Tianjin Medical University General Hospital, Tianjin, China; Tianjin Key Laboratory of Female Reproductive Health and Eugenics, Tianjin Medical University General Hospital, Tianjin, China; Department of Bone and Soft Tissue Tumor, Tianjin Medical University Cancer Institute and Hospital, National Clinical Research Center for Cancer, Key Laboratory of Cancer Prevention and Therapy, Tianjin’s Clinical Research Center for Cancer, Tianjin, China; Department of Breast Imaging, National Clinical Research Center for Cancer, Key Laboratory of Cancer Prevention and Therapy, Tianjin’s Clinical Research Center for Cancer, Tianjin Medical University Cancer Institute and Hospital, Tianjin, China; Department of Public Service Management, School of Management, Tianjin University of Traditional Chinese Medicine, Tianjin, China; Department of Cancer Pharmacology, Tianjin Institute of Medical and Pharmaceutical Sciences, Tianjin Medicine and Health Research Center, Tianjin, China; I.M. Sechenov First Moscow State Medical University (Sechenov University), Moscow, Russia; Department of Bone and Soft Tissue Tumor, Tianjin Medical University Cancer Institute and Hospital, National Clinical Research Center for Cancer, Key Laboratory of Cancer Prevention and Therapy, Tianjin’s Clinical Research Center for Cancer, Tianjin, China; Department of Gynecology and Obstetrics, Tianjin Medical University General Hospital, Tianjin, China; Tianjin Key Laboratory of Female Reproductive Health and Eugenics, Tianjin Medical University General Hospital, Tianjin, China; Department of Bone and Soft Tissue Tumor, Tianjin Medical University Cancer Institute and Hospital, National Clinical Research Center for Cancer, Key Laboratory of Cancer Prevention and Therapy, Tianjin’s Clinical Research Center for Cancer, Tianjin, China

**Keywords:** spatial transcriptomics, tumor microenvironment, bibliometric analysis, transcriptomics

## Abstract

Spatial transcriptomics (ST) integrates spatial data with transcriptomic information, providing high-resolution maps of gene expression within tissue contexts. It has revolutionized studies on cellular function and disease mechanisms, particularly in cancer and immunology. We conducted a bibliometric analysis of 1197 publications from the Web of Science (2015–24), focusing on publication trends, journal distribution, and keyword analysis to identify key research areas in ST. ST publications surged from 2021, with 500 papers in 2023. Five of the top 10 journals are from the Nature Publishing Group. Keyword analysis identified emerging trends like “tumor microenvironment,” “immune infiltration,” and “biomarker,” highlighting ST’s expanding role in cancer and immunology. International collaboration among multidisciplinary teams is crucial for maximizing ST’s potential, and understanding its trends will guide its future impact. Large language models can further enrich the results of bibliometric research, making the findings of bibliometrics more comprehensive and specific.

## Introduction

Spatial transcriptomics (ST) is an advanced technology that integrates spatial information with transcriptomic data, enabling researchers to analyze gene expression within the precise architectural context of tissues [1]. Unlike traditional transcriptomic techniques, ST not only provides information on gene expression levels but also reveals the spatial distribution of genes within tissue samples, thereby offering insights into the complex cellular functions and tissue organization [2]. This technology represents a significant advancement in understanding biological systems, particularly in fields such as tumor microenvironments, neural networks, and developmental processes. By preserving the spatial context of RNA within tissue sections, ST overcomes the limitation of spatial information loss in single-cell transcriptomics, making it a vital tool for exploring cellular heterogeneity and intercellular interactions [3].

The applications of spatial transcriptomics have expanded across multiple research domains, demonstrating unique value in various areas [3–6]. For instance, in cancer research, ST is widely used to decipher the spatial distribution of immune cells within tumors and their interactions with cancer cells, providing new perspectives on tumor heterogeneity and immune infiltration patterns [7, 8]. Additionally, ST has seen growing use in neuroscience, helping researchers identify gene expression differences and cell types across different regions of the brain [9–11]. In the realm of drug discovery and development, the integration of ST has significantly deepened and broadened research efforts by providing spatiotemporal dynamics of drug actions, thereby supporting the development of new therapies and improving treatment strategies [12]. Thus, ST is not only valuable for basic research but also plays a crucial role in personalized medicine, disease prognosis, and the discovery of novel biomarkers.

Bibliometric analysis is a quantitative method for analyzing scientific literature, often used to uncover the overall landscape, development trends, and research hotspots within a specific field [13]. By applying bibliometric analysis, researchers can quantify the volume, impact, and evolution of publications within a domain, providing data-driven insights for strategic planning in scientific research. One of the strengths of bibliometrics is its wide applicability across different disciplines and time periods. It can reveal research hotspots and emerging trends through methods such as keyword co-occurrence analysis and journal distribution analysis. These insights provide valuable decision-making tools for researchers and policymakers, helping them to better navigate research directions and academic trends.

This study aims to not only confirm the rapid expansion of spatial transcriptomics but also highlight its evolving applications in fields that are reshaping clinical practice, especially surgical practice. Based on the analysis of the global research landscape and development trends of the ST field between 2015 and 2024, we focused on hotspot keywords, core journal distribution, and the evolution of research directions within the field of ST. Through these analyses, we hope to provide valuable references for researchers in the field and support future research and international collaboration.

## Material and methods

### Data source

**Figure 1:**
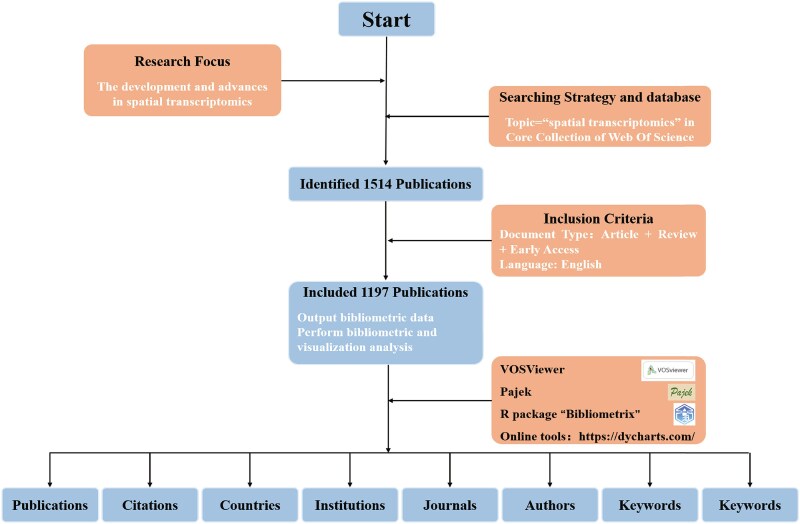
Flowchart of bibliometric analysis on spatial transcriptomics.

In this bibliometric study, the primary data source is the Web of Science (WoS) database, which is widely recognized for its comprehensive coverage of scholarly literature across various disciplines. The study includes literature published from 1 January 2015 to 15 May 2024, focusing on the field of spatial transcriptomics (Fig. 1).

### Data retrieval and collection

The WoS core database was utilized as a data source. The WoS Core Collection was chosen as the primary database for this bibliometric analysis due to its extensive coverage of high-quality, peer-reviewed literature across multiple disciplines. The literature retrieval was conducted using the following search query: (topic = ‘spatial transcriptomics’) AND (language = ‘English’) AND (document type = ‘Articles, Review Articles, Early Access’). Nonpeer-reviewed and non-English documents were excluded. Key bibliographic information such as title, authors, source, publication year, abstract, keywords, citation count, and affiliations was extracted. Data cleaning involved removing duplicates, standardizing names and titles, and addressing missing data to ensure a robust and comprehensive dataset for reliable analysis.

### Clustering and visualization

VOSviewer (1.6.17) was employed for hierarchical clustering and visualization of the bibliometric data. VOSviewer is a powerful tool for constructing and visualizing bibliometric networks, allowing for the identification of clusters and the relationships within the dataset. The visualizations provided by VOSviewer, including network maps and density maps, offered a clear and intuitive representation of the hierarchical relationships and thematic clusters within the ST literature. These visualizations facilitated the identification of key research areas, trends, and potential gaps in the field, providing valuable insights into the structure and dynamics of the research landscape. For clustering analysis, the following cutoff criteria were applied: keywords were included if they appeared at least five times in the dataset, countries were included if they had a minimum of 5 publications, organizations were included if they contributed at least 10 publications, and authors were included if they contributed at least five times. These thresholds were set to ensure that only the most significant terms, countries, institutions, and authors were included in the analysis, while minimizing noise from infrequent terms.

### Scientific production and publication analysis

The analysis of scientific production and publication provides crucial insights into the development and dissemination of research within the field of ST over a continuous period. This analysis involves assessing the volume and patterns of publications to understand the evolution of the field, identify key contributors, and highlight significant trends. Quantitative processing for this analysis was performed with the R package “bibliometrix” (4.0.0). This powerful tool facilitates detailed bibliometric analysis and provides various functions for data manipulation and visualization.

### Using ChatGPT-4o for article analysis

The study focuses on analyzing a collection of research articles, which include both review and original research papers, using a language model Application Programming Interface (API). Data, such as the title and abstract of each article, are extracted from a dataframe containing multiple entries. For each article, a prompt is generated to instruct the model to analyze the research direction based on the article’s title and abstract. All collected “Research Direction,” “Keywords,” “Methods/Techniques,” and “Application Fields” are then consolidated into a single document. This document is subsequently analyzed by ChatGPT-4o-latest. The generated prompt is sent to the language model through an API request, with the model expected to return structured output in JavaScript Object Notation (JSON) format. The model is tasked with summarizing and predicting the key application fields, technical features, technical bottlenecks, and future research directions based on the compiled data. In the event of any errors, the exception is logged, and None is returned. The analysis is performed using Python (version 3.11.9), the OpenAI API, and the Pandas library (version 2.2.2) for data manipulation and communication with the model (Fig. S1).

## Results

### Analysis of the number of publications and citations

**Figure 2:**
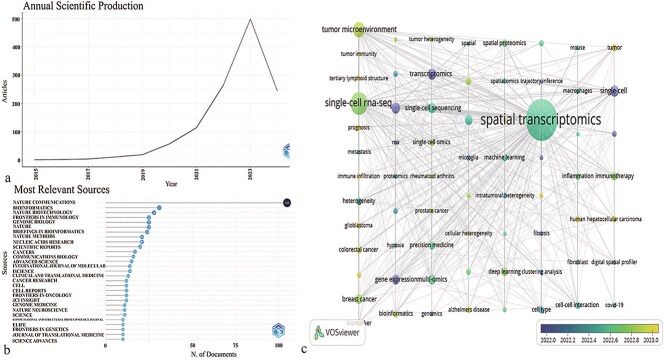
(a) The publication trend; (b) the number of publications in various journals; (c) the key words co-occurrence network analysis.

A total of 1197 publications from 61 countries were included. The earliest study on ST dates back to 2015 and focused on single-cell trajectory analysis in inner ear development and regeneration. Figure 2a suggests the publication trend of papers from 2015 to 2024. The number of publications has generally increased alongside the development of single-cell transcriptomics. Before 2019, the number of published articles was limited. However, since 2021, the annual publication volume has increased significantly, reaching 500 in 2023.

### Analysis of the contributions of journals

A total of 394 journals contributed to the 1197 papers. Figure 2b presents the journals that published >20 papers. *Nature Communications* is the leading journal with 105 publications, accounting for 6.94% of all publications, followed by *Bioinformatics*, *Nature Biotechnology*, and *Frontiers in Immunology*. Among the top 10 journals with the highest number of ST articles, 5 are published by *Nature Publisher*, demonstrating the significant influence of the USA in this field.

### Analysis of keywords

In Fig. 2c, 69 keywords were divided into eight clusters when the minimum number of occurrences was set to five. The top five most frequent keywords were “spatial transcriptomics” (*n* = 393), “single-cell RNA-seq” (*n* = 118), “tumor microenvironment” (*n* = 54), “transcriptomics” (*n* = 30), and “RNA-seq” (*n* = 29). The line between “spatial transcriptomics” and “single-cell RNA-seq” was the thickest, indicating that single-cell RNA-seq has been a crucial tool for spatial transcriptomics over the past 10 years. Meanwhile, “tumor microenvironment,” “tumor immunity,” “tertiary lymphoid structure,” “prognosis,” “immune infiltration,” “biomarker,” and “cellular heterogeneity” emerged as hotspot keywords after 2023. Among all diseases, “glioblastoma,” “colorectal cancer,” “breast cancer,” “rheumatoid arthritis,” “prostate cancer,” “Alzheimer’s disease,” “hepatocellular carcinoma,” and “COVID-19” became hotspots after 2022. These hotspot keywords reveal the broad application of ST in different fields and its integration with various combinative techniques (Table S1).

### Analysis of the contributions of countries

**Figure 3:**
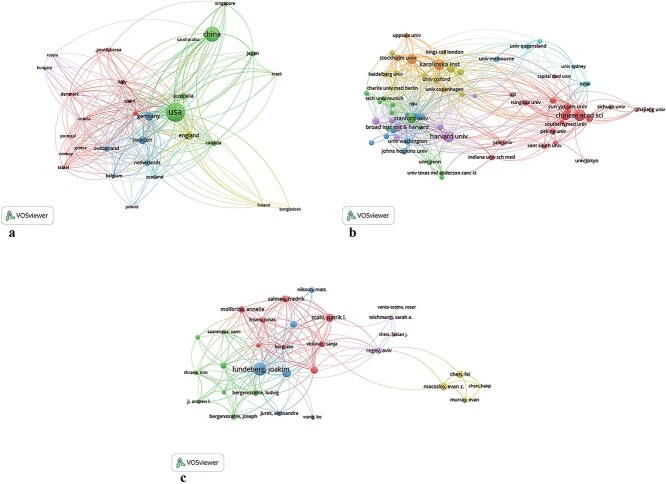
Visualization and analysis of co-authorship of countries (a), organizations (b), and co-authorship (c).

**Figure 4:**
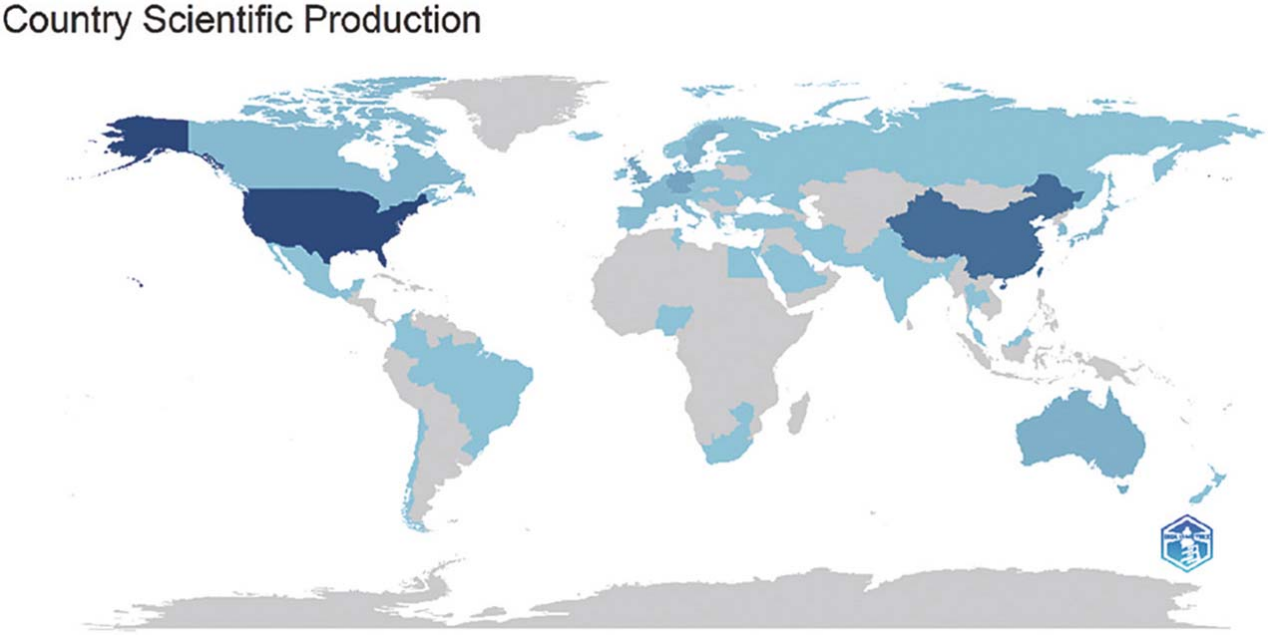
Geographic distribution of global publications on spatial transcriptomics. World map showing the distribution of global publications on spatial transcriptomics.

As shown in Figs 3 and 4, 31 countries were divided into five clusters when the minimum number of publications per country was set to five. The density visualization map of the co-authorship and collaboration network among countries is presented in Fig. 3a. Various colors represent the frequency of co-authorship, with green indicating the highest frequency. The top five countries with the most publications on ST worldwide are the USA (*n* = 553), China (*n* = 351), Germany (*n* = 120), England (*n* = 111), and Sweden (*n* = 97). Among these, the USA (*n* = 408) exhibited the most collaborative relationships, followed by Germany (*n* = 231), England (*n* = 196), China (*n* = 145), and Sweden (*n* = 142). This underscores the significant influence of the USA in the field of ST.

### Analysis of the contributions by institutions

The density visualization map of the organization and collaboration network is shown in Fig. 3b and Table 1. All organizations with a minimum of 10 publications were listed. The top five organizations with the most publications on ST are the Chinese Academy of Sciences (China, *n* = 67), Harvard University (USA, *n* = 63), Karolinska Institute (Sweden, *n* = 56), Royal Institute of Technology (Sweden, *n* = 49), and Stanford University (USA, *n* = 41). Among these organizations, Harvard University (*n* = 55) exhibited the most collaborative relationships, followed by Karolinska Institute (*n* = 45), Royal Institute of Technology (*n* = 39), Chinese Academy of Sciences (*n* = 36), and Stanford University (*n* = 29).

**Table 1:** List of top 10 productive organizations.

**Ranking**	**Institution**	**Country**	**Publication count**	**Citations**	**Collaboration**
1st	Chinese Academy of Sciences	China	67	1027	36
2nd	Harvard university	USA	63	5421	55
3rd	Karolinska institute	Sweden	56	4541	45
4th	Royal Institute of Technology	Sweden	49	5434	39
5th	Stanford university	USA	41	1478	29
6th	Shanghai Jiao Tong University	China	41	2632	21
7th	Broad Institute of MIT & Havard	USA	35	4865	35
8th	Cambridge University	UK	34	2236	28
9th	Michigan University	USA	30	462	13
10th	Massachusetts Institute of Technology	USA	26	1759	25

### Analysis of contributions of the authors

The density visualization map of the authors is shown in Fig. 3c and Table 2. All authors with a minimum of 10 publications were listed. The top five authors with the most publications on ST are Joakim Lundeberg (Sweden, *n* = 39), Ludvig Larsson (Sweden, *n* = 18), Patrik L. Ståhl (Sweden, *n* = 14), Evan Z. Macosko (USA, *n* = 13), and José Fernández Navarro (Spain, *n* = 12). Among these authors, Joakim Lundeberg (*n* = 180) exhibited the most collaborative relationships, followed by Ludvig Larsson (*n* = 94), Patrik L. Ståhl (*n* = 74), Evan Z. Macosko (*n* = 44), and José Fernández Navarro (*n* = 59). The node size was positively correlated with the number of publications of a certain author and line thickness between nodes represented the cooperation frequency.

**Table 2:** List of top 10 productive authors.

**Ranking**	**Author**	**Country**	**Publication count**	**Citation**	**Collaboration**
first	Joakim Lundeberg	Sweden	39	5255	180
2nd	Ludvig Larsson	Sweden	18	1438	94
3rd	Patrik L. Ståhl	Sweden	14	2703	74
4th	Evan Z. Macosko	USA	13	1263	44
5th	José Fernández Navarro	Spain	12	2850	59
6th	Chen Fei	China	12	1032	37
7th	Stefania Giacomello	Sweden	11	1936	40
8th	Fredrik Salmen	Netherlands	11	3009	73
9th	Annelie Mollbrink	Sweden	10	2265	50
10th	Aviv Regev	USA	10	1181	40

### Analysis of citations and highly cited study

**Figure 5:**
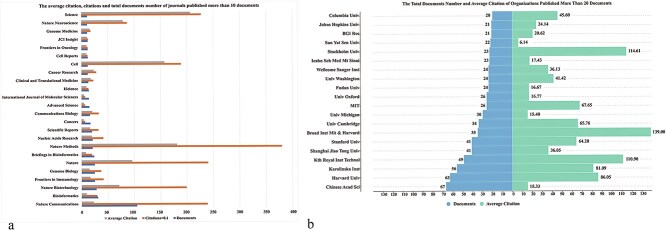
(a) The average citation, citations, and total documents number of journals published >10 documents; (b) the total documents number and average citation of organization published >20 documents.

**Figure 6:**
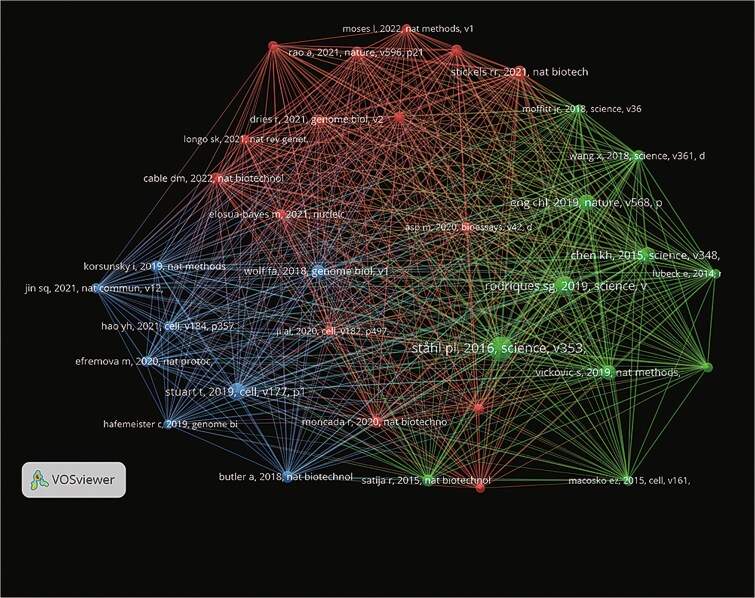
The network map of highly cited references (>100 times) with three clusters.

The number of documents published, total citations, and average citations per document are compared across various journals (Fig. 5 & Table 3). High total and average citations highlight the significant impact of *Cell* and *Nature*. Additionally, *Science*, *Nature Neuroscience*, and *Genome Medicine* demonstrate strong performance in these metrics, underscoring their prominence in their respective fields. The analysis of institutions reveals the Chinese Academy of Sciences as the top publisher with 67 documents. Harvard University and Karolinska Institute show robust performance in both publication count and impact. The Broad Institute of Massachu-setts Institute of Technology (MIT) and Harvard stands out with an impressive average citation, reflecting the high quality and significant influence of its research. Out of 53 628 references, 34 articles were cited >100 times, as shown in the cluster analysis (Fig. 6). The visualization highlights three distinct clusters: red, green, and blue, indicating strong cocitation relationships. This clustering demonstrates the interconnectedness and the pivotal contributions of these highly cited articles in advancing spatial transcriptomics (ST) research.

**Table 3:** List of top 10 most cited papers on spatial transcriptomics.

**Ranking**	**First author**	**Title**	**Journals**	**Publication year**	**Total citations**
1st	KORSUNSKY I	Fast, sensitive, and accurate integration of single-cell data with Harmony	NAT METHODS	2019	2325
2nd	STÅHL PL	Visualization and analysis of gene expression in tissue sections by spatial transcriptomics	SCIENCE	2016	1402
3rd	STARK R	RNA sequencing: the teenage years	NAT REV GENET	2019	891
4th	VICKOVIC S	High-definition spatial transcriptomics for in situ tissue profiling	NAT METHODS	2019	509
5th	WU SZ	A single-cell and spatially resolved atlas of human breast cancers	NAT GENET	2021	423
6th	BACCIN C	Combined single-cell and spatial transcriptomics reveal the molecular, cellular and spatial bone marrow niche organization	NAT CELL BIOL	2020	418
7th	MONCADA R	Integrating microarray-based spatial transcriptomics and single-cell RNA-seq reveals tissue architecture in pancreatic ductal adenocarcinomas	NAT BIOTECHNOL	2020	414
8th	RAO A	Exploring tissue architecture using spatial transcriptomics	NATURE	2021	397
9th	STICKELS RR	Highly sensitive spatial transcriptomics at near-cellular resolution with Slide-seqV2	NAT BIOTECHNOL	2021	369
10th	CHEN WT	Spatial Transcriptomics and In Situ Sequencing to Study Alzheimer’s Disease	CELL	2020	355

### Comprehensive report from large language model

Artificial intelligence (AI)–driven analysis of the collected data provides insights into the key application fields, technical features, bottlenecks, and future research directions for spatial transcriptomics. The analysis highlights cancer biology and immunotherapy, neuroscience and neurological disorders, and developmental biology as the leading application fields (Table S2). High-resolution spatial profiling techniques, such as MERFISH and Slide-SeqV2, enable near-cellular resolution and enhance gene expression mapping. AI tools, like deep learning models and spatial clustering algorithms, improve the analysis of complex biological data (Table S3). However, challenges remain, including balancing high spatial resolution with throughput, addressing RNA dropout in imaging methods, and integrating data across platforms (Table S4). There is also a need to better contextualize spatial gene expression and translate findings from model organisms to human diseases. Future directions suggest a focus on enhanced spatial resolution techniques, such as subcellular and organelle-level analysis. The integration of multi-omics data, particularly combining spatial transcriptomics with proteomics and metabolomics, is expected. AI tools, including generative models and explainable AI, will improve predictions and understanding of gene networks (Table S5).

## Discussion

This study provides a comprehensive bibliometric analysis of 1197 publications related to ST from the WoS Core Collection database, spanning the years 2015–24. The analysis reveals key trends, emerging hotspots, and the global distribution of research efforts in the ST field. The results demonstrate that ST, as an emerging technology, has seen significant development and widespread application in recent years. These findings not only underscore the importance of ST in the scientific community but also offer valuable insights for future research directions in this field.

**Figure 7:**
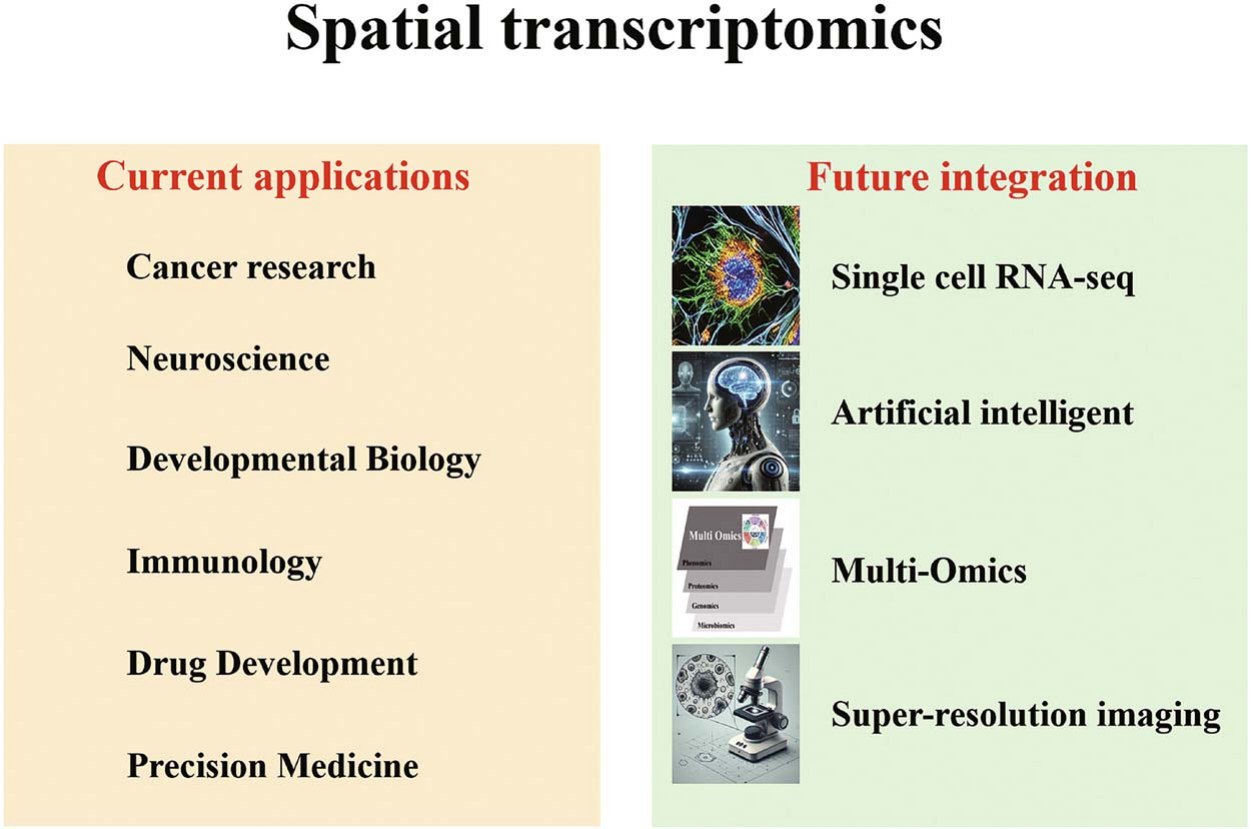
Summary of spatial transcriptomics in current applications and future integration.

ST has been proven to be highly valuable across various research fields (Fig. 7). In cancer research, ST reveals the spatial distribution of cells and gene expression within the tumor microenvironment, offering new insights into tumor heterogeneity and immune infiltration, which are crucial for personalized therapy and biomarker discovery [14, 15]. In neuroscience, ST helps to map gene expression patterns across different brain regions, providing a deeper understanding of neurological diseases such as Alzheimer’s by highlighting specific gene expression changes in affected areas [16, 17]. In developmental biology, ST tracks the spatiotemporal dynamics of gene expression during embryonic development, uncovering the mechanisms behind cell fate decisions and tissue differentiation at various developmental stages, which is vital for studying developmental abnormalities and congenital diseases [18]. In immunology, ST technology enhances our understanding of how immune cells are spatially organized within tissues and their roles in immune responses, particularly in their interactions with pathogens or tumor cells [19, 20]. Moreover, ST is widely applied in drug development and precision medicine, where it aids in optimizing drug design, improving therapeutic outcomes, and discovering new biomarkers by analyzing gene expression changes in tissues following drug treatment [12, 21]. Overall, ST technology, with its ability to reveal spatial heterogeneity in gene expression, is driving the forefront of research in oncology, neuroscience, developmental biology, immunology, and precision medicine. It not only offers new tools for basic research but also opens up new possibilities for clinical applications. As the technology continues to advance, ST is expected to have an even greater impact across more fields.

The analysis of publication numbers and citation trends shows a steady increase in the number of ST-related publications since the first studies appeared in 2015. Particularly after 2021, there has been a sharp rise in publications, reaching a peak of 500 papers in 2023. This trend indicates that as single-cell transcriptomics technology has advanced, ST research has concurrently gained momentum, becoming a powerful tool for elucidating the spatial heterogeneity of gene expression within tissues. The development of this technology has provided new perspectives for many research fields, especially in tumor microenvironments, immunology, and neuroscience [22, 23]. The citation analysis reveals that high-impact journals such as *Nature Communications* and *Bioinformatics* have published a substantial number of ST articles, highlighting the academic value of ST and the widespread recognition of its research quality. The rapid growth of ST stems from several key technological breakthroughs. High-resolution ST platforms, such as spatially resolved RNA sequencing, have allowed researchers to create more detailed and accurate spatial maps of gene expression [24]. The integration of ST with multi-omics approaches has further enhanced its potential by combining ST with proteomics, metabolomics, and other molecular layers [25] [40532697]. The use of AI and machine learning (ML) in analyzing large-scale spatial data is accelerating progress in the field. However, the spatial resolution of ST is limited by the sensitivity of current platforms, making it difficult to resolve fine-scale tissue structures. Integrating multi-omics data is hindered by differences in data formats and computational issues [26] [40154487]. Analyzing large spatial transcriptomic datasets also requires significant computational power and efficient data management [40651849]. These challenges must be addressed to fully unlock ST’s potential in biomedical research and clinical applications.

The analysis of journal contributions shows that leading journals in ST research include *Nature Communications*, *Bioinformatics*, *Nature Biotechnology*, and *Frontiers in Immunology*. These journals have published a significant proportion of ST-related literature, with five of the top 10 journals being from the Nature Publishing Group, indicating the strong influence of the USA in this field. The concentration of ST publications in these journals reflects the interdisciplinary nature of ST technology and its critical role in advancing life sciences research, particularly in deciphering the distribution of immune cells within tumor microenvironments and the interactions between cells.

The keyword analysis highlights the research hotspots and application areas within ST. The most frequent keywords include “spatial transcriptomics,” “single-cell RNA-seq,” “tumor microenvironment,” “transcriptomics,” and “RNA-seq.” The close association between these keywords suggests that single-cell RNA-seq has played a pivotal role in the development of ST technology, particularly in analyzing cellular heterogeneity and tissue structure. Additionally, keywords such as “tumor microenvironment,” “tumor immunity,” “tertiary lymphoid structure,” “prognosis,” “immune infiltration,” “biomarker,” and “cellular heterogeneity” have emerged as hotspots since 2023, reflecting the broad application of ST in cancer research and disease prognosis. The research on diseases like “glioblastoma,” “colorectal cancer,” “breast cancer,” “rheumatoid arthritis,” “prostate cancer,” “Alzheimer’s disease,” “hepatocellular carcinoma,” and “COVID-19” further demonstrates the potential of ST technology in various disease studies [16, 27–33]. The emergence of these keywords indicates that ST is increasingly being integrated with other technologies, thereby expanding its research scope.

In terms of country contributions, the USA, China, Germany, England, and Sweden are the primary contributors to ST research. The USA leads in both publication numbers and international collaborations, indicating its dominant position in the ST field. China, as an emerging research power, has shown strong potential, particularly in international collaboration and scientific output. European countries such as Germany, England, and Sweden also play significant roles in ST research, especially in methodological innovation and technological application.

Institutional contribution analysis shows that the Chinese Academy of Sciences, Harvard University, Karolinska Institute, Royal Institute of Technology (Sweden), and Stanford University are the leading institutions in ST research. These institutions not only lead in publication numbers but also excel in international collaborations. Notably, Harvard University and Karolinska Institute, with their extensive collaborative networks and high-impact research outputs, further establish their leadership in ST research. The work of these institutions has not only advanced ST technology but also facilitated its application in disease research and drug discovery.

Author contribution analysis identifies Joakim Lundeberg, Ludvig Larsson, Patrik L. Ståhl, Evan Z. Macosko, and José Fernández Navarro as leading authors in ST research. Joakim Lundeberg stands out with his extensive collaborative relationships and prolific research output, establishing him as a key figure in the field [16, 34–38]. The contributions of these top authors are significant not only in quantity but also in quality, playing a crucial role in the advancement of ST research.

The analysis of citation counts and highly cited papers further confirms the importance of journals like *Cell* and *Nature* in ST research. The highly cited papers published in these journals reflect the breadth and depth of research in this field and highlight the impact of ST technology in scientific research. Papers with over 100 citations have played a landmark role in ST research, with their cocitation networks illustrating the pivotal contributions of these studies.

ST represents a groundbreaking advancement in genomics, combining spatial context with gene expression profiling [39, 40]. Among the top works in the field, several key studies have made substantial scientific contributions, driving both technological development and clinical applications. A seminal study by Ståhl *et al*. introduced high-definition ST, enabling the detailed mapping of gene expression across tissue sections [34]. This breakthrough has since become foundational, particularly in cancer research, where Pelka *et al*. uncovered spatially organized immune hubs in colorectal cancer. Such insights have significantly advanced our understanding of tumor heterogeneity and immune infiltration patterns, which are crucial for developing personalized therapies [8]. Furthermore, in the realm of neuroscience, Chen *et al*. leveraged ST to identify region-specific gene expression changes in Alzheimer’s disease, offering new directions for therapeutic targets [16]. In addition, ST has proven valuable in developmental biology, as demonstrated by Sampath *et al*., who mapped gene expression during early organogenesis, revealing critical mechanisms underlying tissue differentiation [18]. These landmark studies highlight the qualitative impact of ST beyond publication volume, as they provide the basis for transformative applications in personalized medicine, biomarker discovery, and the understanding of complex disease mechanisms. Collectively, the body of our research within this field underscores the potential of ST to deliver high-impact findings, with its continued evolution promising even greater contributions across diverse biological domains.

ST is poised to achieve broader and deeper applications by integrating with other cutting-edge technologies, driving significant advancements in life sciences and medical research (Fig. 7). Combining ST with single-cell RNA sequencing (scRNA-seq) will enhance our understanding of the spatial distribution and functional roles of cells within complex tissues, such as the brain, heart, and tumors [28, 41]. The incorporation of AI and ML into ST data processing will further streamline analysis, enabling the identification of complex gene expression patterns and predicting cell interactions, ultimately advancing personalized medicine [42, 43]. Recent advancements in single-cell ST allow high-resolution gene expression analysis at the single-cell level, improving our understanding of tissue architectures. Models like SiGra and xSiGra combine high-resolution imaging and transcriptomic data to enhance spatial characterization. These graph-based transformer models improve spatial domain identification and provide interpretability, offering insights into the roles of specific genes and cells in tissues. These advancements address challenges like data sparsity and noise, enabling a more accurate study of cellular interactions and heterogeneity, especially in diseases like cancer [44, 45]. Additionally, the integration of ST with multi-omics approaches, including genomics, proteomics, and metabolomics, will allow researchers to explore molecular interactions within their spatial context, building comprehensive disease networks and advancing systems biology [46, 47]. Pairing ST with high-resolution imaging technologies, such as light-sheet microscopy and super-resolution microscopy, will facilitate the simultaneous analysis of gene expression and tissue structure, offering new insights into the relationship between gene regulation and tissue function [48, 49]. Despite its vast potential, ST still faces challenges in spatial resolution, data complexity, and cost, necessitating improvements in these areas [50, 51]. These challenges are particularly relevant in the context of solid organ transplantation, where spatially resolved gene expression data could provide crucial insights into graft rejection, immune responses, and tissue regeneration. For instance, ST could be used to map the molecular landscape of transplant tissues, identifying early biomarkers of rejection or tolerance [52]. Additionally, ST could help in understanding the dynamics of immune cell infiltration and interactions within the transplant microenvironment, offering opportunities for more precise therapeutic interventions [53]. Moving forward, ST’s collaboration with these innovative technologies will propel biological research and medical applications to new heights, bringing transformative breakthroughs in personalized medicine and disease treatment.

ST holds transformative potential for surgical applications, particularly in the realm of precision surgery and intraoperative decision-making [54]. One of the most impactful contributions of ST is its ability to map the spatial distribution of gene expression within tissues, providing insights into tumor heterogeneity, immune cell infiltration, and tissue microenvironment dynamics. These capabilities are critical for surgical oncology, where understanding the precise boundaries of tumor margins can be the difference between successful resection and recurrence. Recent advancements have demonstrated how ST can be applied in presurgical planning, enabling surgeons to identify not only the anatomical extent of a tumor but also its molecular characteristics, thereby refining surgical strategies and potentially reducing the need for follow-up procedures. For instance, Pelka *et al*. showed that spatial immune hubs within colorectal cancer tissues could serve as indicators of tumor aggressiveness and response to immunotherapy, directly informing surgical resection strategies and perioperative treatments [8]. Furthermore, ST’s integration with other spatial omics technologies provides a detailed molecular atlas of tissue architecture, which can guide the development of personalized treatment plans in real time. In neurosurgery, ST has been pivotal in identifying molecular signatures in brain tumors, helping surgeons avoid critical functional areas during resection, thereby reducing postoperative neurological deficits [27, 55, 56]. Additionally, the application of ST in transplant surgery has begun to uncover molecular signals associated with tissue rejection and immune compatibility, offering new biomarkers that could improve graft survival [52, 57, 58]. As ST technologies continue to advance, their translation into surgical practice will likely extend beyond oncology and neurology, contributing to the broader field of regenerative medicine and reconstructive surgery by offering new insights into tissue repair and healing dynamics. These developments highlight the clinical relevance of ST not only in research but also as a cutting-edge tool that bridges molecular insights with surgical innovation, driving more precise and personalized patient care.

The AI-driven analysis leverages large language models, such as ChatGPT-4o, to synthesize extensive bibliographic and content data, offering a more comprehensive and forward-looking perspective on spatial transcriptomics. Unlike bibliometric analysis, which focuses on counting publications and identifying keywords, the AI model provides insights into how various research areas are interconnected and predicts future trends. For example, it highlights the growing integration of spatial transcriptomics with multi-omics technologies, particularly proteomics and metabolomics, as a key direction for future research [59]. Additionally, the AI model predicts that enhancing spatial resolution techniques, such as subcellular and organelle-level analysis, will be crucial for advancing the field [60]. These insights stem from the model’s ability to process complex data relationships, offering a nuanced understanding of how ST is likely to evolve.

## Limitation

Despite providing valuable insights into the trends and developments in ST research, this study has certain limitations. Firstly, it relies solely on the WoS Core Collection database, which, while authoritative, may exclude relevant studies available in other databases such as PubMed and Scopus. This limitation could impact the comprehensiveness of the findings. Secondly, bibliometric analysis primarily focuses on publication counts and citation metrics, which, while useful for identifying trends, may not fully capture the quality and innovativeness of the research. Some studies with significant scientific value might be underrepresented due to shorter publication times or niche areas. Thirdly, it is important to note that our study is limited to English-language publications, which may bias the country-specific analysis. Non-English literature, which could contain relevant studies from regions with different publication trends, was not included in this study. Future work could benefit from expanding the analysis to include publications in other languages, potentially leading to a more comprehensive understanding of global trends in spatial transcriptomics. Fourthly, VOSviewer and bibliometrix were selected for their strong capabilities in visualizing bibliometric networks and performing advanced clustering analysis. VOSviewer handles author name disambiguation by using a standardized author name format, though we acknowledge that some name ambiguities may persist, particularly for common surnames. Data cleaning steps included removing duplicates, standardizing author names, and addressing missing data to ensure the robustness and reliability of the dataset. These tools are widely used in bibliometric analysis, but it is important to note that they have some limitations in dealing with inconsistencies in author names and institutional affiliations. While the AI-driven analysis provides valuable qualitative insights, it is important to acknowledge its limitations. The accuracy of the model’s predictions depends on the quality and breadth of the data it processes, as well as the training it has received. Thus, combining both bibliometric and AI-driven analyses offers a more robust and comprehensive view of the field. Bibliometric analysis provides data-driven, quantifiable insights, while AI-driven analysis adds deeper qualitative interpretation and predictions, enabling a more holistic understanding of the research landscape. Finally, the study’s analysis is current only up to 2024, and the rapidly evolving nature of ST technology means that future trends may differ, necessitating updates to this analysis to maintain its relevance.

## Conclusion

This bibliometric study systematically reviewed the global research landscape of spatial transcriptomics from 2015 to 2024. The analysis revealed that ST has experienced rapid growth since its introduction in 2015, with a significant increase in publications beginning in 2021, reaching a peak in 2023. This trend reflects the growing recognition and application of ST in biomedical research. The leading contributors to this field include the USA, China, Germany, the UK, and Sweden, with the USA dominating in both publication volume and international collaboration. High-impact journals such as *Nature Communications* and *Bioinformatics* have published numerous ST-related articles, underscoring the importance and influence of this technology. Keyword analysis identified “tumor microenvironment,” “single-cell RNA-seq,” and “immune infiltration” as research hotspots, indicating the promising applications of ST in cancer research and immunology. Additionally, leading research institutions like the Chinese Academy of Sciences, Harvard University, and Karolinska Institute have made significant contributions, demonstrating their pivotal roles in advancing ST technology. Large language models can further enrich the results of bibliometric research, making the findings of bibliometrics more comprehensive and specific. Overall, this study highlights the global research landscape of ST, providing valuable insights and reference points for future research directions.

**Key Points**

- This study presented a comprehensive bibliometric analysis of spatial transcriptomics in the past 10 years.
- Using leading analytical methods, key trends, influential contributors, and emerging themes were identified on spatial transcriptomics.
- Single-cell RNA-seq integration, tumor microenvironment studies, and diverse disease applications were key research areas in the field of spatial transcriptomics.

## Supplementary Material

Supplement_Material_bbaf553

## Data Availability

The datasets supporting the conclusions of this article are available in the Web of Science Core Collection database.

## Artificial intelligence tools

No application.
