# Supplementary material for: Spatial transcriptomics: a bibliometric analysis with large language model on English literatures
Source: Brief Bioinform. 2025 Oct 26;26(5):bbaf553. doi: 10.1093/bib/bbaf553 (PMC12554094; doi:10.1093/bib/bbaf553)
Supplement: Supplement_Material_bbaf553 [file supplement_material_bbaf553.pdf]

Supplement Material

```
def send_request_to_llm(prompt):
    try:
        json_result = client.chat.completions.create(
            model='chatgpt-4o',
            messages=[{'role': 'user', 'content': prompt}],
            stream=False
        )
        return json_result.choices[0].message.content
    except Exception as e:
        print(f"Requests failed: {e}")
        return None
```

Figure S1: The code of automated research analysis using ChatGPT-4o.

Alt Text: Figure S1 shows the code for automated research analysis using ChatGPT-4o. It includes a function that sends a request to the model with a user-provided prompt and retrieves the response for further analysis.

Table S1: The top 69 keywords with the highest frequency

NO.	keyword
1	spatial transcriptomics
2	gene-expression
3	expression
4	seq
5	tissue
6	single-cell
7	atlas
8	cells
9	identification
10	cancer
11	genome-wide expression
12	RNA
13	heterogeneity
14	RNA-seq
15	visualization
16	reveals
17	single-cell RNA sequencing
18	architecture
19	activation
20	tumor microenvironment
21	mouse
22	transcriptomics
23	cell
24	landscape
25	differentiation
26	macrophages
27	t-cells

28	immunotherapy
29	stem-cells
30	growth
31	mechanisms
32	survival
33	progression
34	protein
35	gene
36	organization
37	inflammation
38	reconstruction
39	proteins
40	scRNA-seq
41	diversity
42	disease
43	dynamics
44	microenvironment
45	microglia
46	gene expression
47	b-cells
48	brain
49	fibroblasts
50	receptor
51	breast cancer
52	evolution
53	cortex
54	migration
55	model
56	proliferation

57	resolution
58	chemotherapy
59	risk
60	single
61	breast-cancer
62	injury
63	localization
64	metastasis
65	pathway
66	prognosis
67	single cells
68	single-cell transcriptomics
69	spatial transcriptome

Table S2: A Comprehensive Report on Key Application Fields in Spatial Transcriptomics Using ChatGPT-4o

Cancer Biology and Immunotherapy	● Tumor Microenvironment (TME) Heterogeneity: Mapping spatial gene expression in cancers (e.g., breast, prostate, pancreatic ductal adenocarcinoma, glioblastoma) to identify tumor subpopulations, immune cell infiltration, and stromal interactions.<br>● Immunotherapy Biomarkers: Using spatial transcriptomics to identify immune cell spatial organization (e.g., TAMs, T cells) and molecular signatures (e.g., SPP1, CD8+ T cell dynamics) for predicting immunotherapy response.<br>● Metastasis Mechanisms: Studying spatial heterogeneity in metastatic niches (e.g., breast cancer in lymph nodes, prostate cancer in bone) to uncover metastasis drivers and therapeutic targets.<br>● Therapeutic Resistance: Investigating spatially resolved metabolic reprogramming (e.g., glycolysis to OXPHOS transitions) and stromal-immune crosstalk in drug-resistant tumors.<br>● Lymphoid Structures: Characterizing tertiary lymphoid structures (TLSs) in cancers (e.g., renal cell carcinoma, lung cancer) to improve immunotherapy strategies.

Neuroscience and Neurological Disorders	● Brain Development and Evolution: Decoding spatiotemporal gene expression in the cerebral cortex, hippocampus, and spinal cord to understand neurodevelopmental programs and evolutionary divergence.<br>● Neurodegenerative Diseases: Analyzing spatial gene networks in Alzheimer's (e.g., TREM2+ macrophages, mitochondrial dysfunction), Parkinson's (dopaminergic neuron heterogeneity), and multiple sclerosis (microglial heterogeneity).<br>● Neural Circuits and Plasticity: Mapping synapse-specific gene expression and neuronal diversity (e.g., sensory neurons, interneurons) to study functional connectivity and plasticity.<br>● Neuroinflammation: Investigating immune-glia crosstalk (e.g., astrocyte subtypes, microglial signaling) in diseases like ALS and MS.<br>● Neurological Regeneration: Studying spatial transcriptomic changes in scar formation (e.g., glial scarring post-MI) and regenerative niches (e.g., spinal cord injury models).

Developmental and Regenerative Biology	● Embryonic Development: Profiling spatial gene expression in organs (e.g., heart, lung, liver) to elucidate morphogenesis, germ layer specification, and developmental disorders.<br>● Organoid and In Vitro Models: Leveraging spatial transcriptomics to study organoid maturation (e.g., cerebral organoids, intestinal organoids) and validate developmental mechanisms.<br>● Tissue Repair and Fibrosis: Investigating spatial heterogeneity in wound healing (e.g., liver, lung, muscle) to identify fibroblast subtypes and regenerative pathways.<br>● Stem Cell Niches: Characterizing spatial organization of stem cells (e.g., hematopoietic stem cells, intestinal crypts) and their interactions with the microenvironment.<br>● Aging and Senescence: Analyzing age-related changes in tissue architecture (e.g., kidney, liver, brain) to uncover mechanisms of degeneration and rejuvenation.

Immune and Inflammatory Diseases	● Autoimmune Pathogenesis: Studying immune cell spatial dynamics in RA, psoriasis, and spondyloarthritis to identify inflammatory niches and therapeutic targets.<br>● Infectious Diseases: Profiling immune responses to pathogens (e.g., SARS-CoV-2, malaria) in infected tissues to understand host-pathogen interactions.<br>● Organ-Specific Immunity: Mapping immune cell distribution in tissues (e.g., liver, lung, kidney) to study localized immune responses and disease mechanisms.<br>● Immunometabolism: Investigating metabolic reprogramming in immune cells (e.g., macrophages, T cells) during inflammation and disease.<br>● Immunotherapy Optimization: Using spatial data to design spatially targeted therapies (e.g., TAM inhibition, B cell modulation).

Plant and Organismal Biology	● Plant Development: Studying spatial gene expression in shoot apical meristems, root development, and vascular tissues to understand plant morphogenesis.<br>● Microbial Interactions: Profiling spatial heterogeneity in plant-microbe symbiosis (e.g., mycorrhizae) and microbial pathogenesis.<br>● Crop Improvement: Identifying tissue-specific gene networks in crops (e.g., maize, soybean) to enhance yield and stress resistance.<br>● Comparative Genomics: Using spatial transcriptomics to compare developmental programs across species (e.g., mouse vs. human, plants vs. animals).<br>● Plant Immunity: Decoding spatial immune responses in crops to pests and pathogens (e.g., viral infections, fungal resistance).

Table S3: A Comprehensive Report on Technical Features and Innovations in Spatial Transcriptomics Using ChatGPT-4o

High-Resolution Spatial Profiling	● MERFISH and Slide-SeqV2: Near-cellular resolution techniques for RNA detection in tissues.<br>● Multiplexed Imaging: Combinatorial fluorescence and spectral encoding for simultaneous mRNA and protein profiling.<br>● In Situ Sequencing: Methods like Padlock probes for high-throughput, single-molecule-level transcriptomics.<br>● 3D Tissue Modeling: Combining spatial transcriptomics with 3D imaging (e.g., kidney, liver, brain) to map gene expression in three dimensions.<br>● Microfluidic Platforms: High-throughput systems for spatially barcoded RNA capture and analysis.

Computational and AI-Driven Analysis	● Deep Learning for Deconvolution: Tools like TANGRAM and BULK2SPACE to infer cell-type composition from bulk RNA-seq and spatial data.<br>● Spatial Clustering Algorithms: Graph-based methods (e.g., STAA, STEEL) to identify tissue domains and cellular interactions.<br>● Multi-Omics Integration: Combining spatial transcriptomics with proteomics, epigenomics, and metabolomics for holistic profiling.<br>● Bayesian and Probabilistic Models: Frameworks for gene expression prediction, spatial pattern detection, and uncertainty quantification.<br>● Data Harmonization: Algorithms to align spatial transcriptomics datasets across platforms and tissues.
Novel Experimental Protocols	● Barcoded Microarrays: High-density spatial capture methods for plant and mammalian tissues.<br>● Optical Tagging: Retaining spatial information in multicellular samples using optical cell tagging.<br>● Frozen Tissue Optimization: Protocols to preserve RNA integrity in FFPE and frozen tissues.

	- Live Cell Tracking: Methods like SPACECAT for real-time spatial transcriptomics in live cells.<br>- Cost-Effective Solutions: Miniaturized and scalable techniques (e.g., POLONY Gels, CLAMPFISH) to reduce experimental costs.

Table S4: A Comprehensive Report on Technical Bottlenecks and Challenges in Spatial Transcriptomics Using ChatGPT-4o

Spatial Resolution and Throughput	- Single-Cell vs. Tissue-Level Resolution: Balancing high-resolution gene detection with large-scale tissue coverage.<br>- Dropout Events: Addressing low RNA detection efficiency in imaging-based methods.<br>- Artifact Correction: Resolving issues like spot swapping and off-target signals in spatial data.<br>- Sample Preparation: Optimizing protocols for RNA preservation in complex tissues (e.g., brain, liver).<br>- Throughput Limitations: Scaling up spatial transcriptomics for high-throughput clinical and agricultural applications.

Data Integration and Interpretation	● Multi-Omics Alignment: Integrating spatial transcriptomics with single-cell RNA-seq, proteomics, and epigenomics data.<br>● Cross-Platform Compatibility: Standardizing data formats and normalization pipelines for reproducibility.<br>● Biological Contextualization: Linking spatial gene expression to functional annotations (e.g., ligand-receptor interactions).<br>● Computational Complexity: Handling large datasets with sparse gene expression and high-dimensional spatial coordinates.<br>● Interpretability Gaps: Decoding spatial patterns into mechanistic insights (e.g., gene regulatory networks, cell-cell communication)

Biological and Clinical Relevance	● Dynamic Tissue States: Capturing temporal changes (e.g., development, disease progression) in spatial transcriptomic profiles.<br>● Heterogeneous Cell Types: Resolving rare or transient cell populations (e.g., CAFs, senescent cells).<br>● Species-Specific Differences: Translating findings from model organisms (e.g., mice, zebrafish) to human diseases.<br>● Clinical Biomarker Discovery: Validating spatial signatures for prognosis and treatment response in cancers and inflammatory diseases.<br>● Ethical and Regulatory Hurdles: Addressing challenges in human tissue studies and data sharing for precision medicine.

Table S5: A Comprehensive Report on Predictions for Future Research Directions in Spatial Transcriptomics Using ChatGPT-4o

Enhanced Spatial Resolution and Multi-Scale Profiling	● Subcellular and Organelle-Level Profiling: Techniques like spatial-ATAC-seq and SEAM will map chromatin accessibility and metabolic heterogeneity at subcellular scales.<br>● 3D Spatial Transcriptomics: Combining expansion microscopy with RNA capture to study 3D tissue architecture (e.g., liver lobules, brain circuits).<br>● Real-Time Spatial Profiling: Development of in vivo spatial transcriptomics for dynamic gene expression tracking in living organisms.
Multi-Omics Integration and Systems Biology	● Epigenome-Transcriptome Correlation: Joint profiling of DNA methylation and RNA expression in spatial contexts (e.g., cancer, neurodevelopment).<br>● Metabolome-Transcriptome Linkage: Spatial metabolomics tools will integrate with transcriptomics to study metabolic reprogramming in tumors and immune cells.<br>● Proteogenomic Mapping: Combining spatial proteomics with transcriptomics to validate gene expression and protein interactions in tissues.

AI and Machine Learning for Data Analysis	● Explainable AI Models: Tools like TALKIEN and REDECONVE will prioritize interpretable predictions of gene networks and cell interactions.<br>● Generative Models: GANs and VAEs will simulate spatial transcriptomic data to augment training for deconvolution and biomarker discovery.<br>● Automated Pipelines: Development of user-friendly platforms (e.g., SODB, Quantish) to streamline data processing and visualization for non-experts.
Clinical Translation and Precision Medicine	● Immunotherapy Biomarkers: Spatial signatures of immune cell localization (e.g., CD8+ T cell density, TLS formation) will guide personalized immunotherapy.<br>● Early Disease Detection: Spatial transcriptomics will enable early identification of precancerous lesions (e.g., IPMNs, Barrett's esophagus) and subclinical inflammation.<br>● Therapeutic Targeting: Spatial maps of CAFs, TAMs, and other stromal cells will inform spatially targeted therapies (e.g., anti-SPP1, anti-POSTN strategies).

Addressing Technical and Biological Limitations	● Standardization of Protocols: Consensus on RNA extraction, library preparation, and data normalization to reduce batch effects.<br>● Cost-Effective Technologies: Development of scalable methods (e.g., CLAMPFISH, miniature-ST) to enable widespread adoption in low-resource settings.<br>● Longitudinal Studies: Combining spatial transcriptomics with longitudinal imaging to track disease progression and treatment effects.<br>● Cross-Species Models: Leveraging zebrafish, mouse, and human data to validate developmental and disease mechanisms.<br>● Ethical and Inclusive Research: Expanding spatial transcriptomics to diverse populations to address disparities in disease mechanisms and treatment outcomes.

Emerging Applications in Non-Traditional Fields	• Environmental and Agricultural Biology: Spatial transcriptomics will study plant-microbe interactions and crop stress responses.<br>• Neuroprosthetics and Bioengineering: Mapping neural circuits and immune responses in engineered tissues for advanced neuroprosthetics.<br>• Evolutionary Biology: Decoding spatial gene expression in non-model organisms to understand evolutionary adaptations.<br>• Synthetic Biology: Spatial data will guide the design of synthetic tissues and organoids with precise spatial gene regulation.<br>• Digital Pathology: Integration with histopathology to create AI-driven diagnostic tools (e.g., predicting chemoresistance in PDAC).
